# DDFU-Net: A Deep Decoder-Focused U-Net Model for Retinal Lesion Segmentation

**DOI:** 10.1007/s10439-026-04032-w

**Published:** 2026-03-12

**Authors:** María Herrero-Tudela, Roberto Romero-Oraá, Gonzalo C. Gutiérrez-Tobal, Roberto Hornero, María I. López, Pedro Romero-Aroca, María García

**Affiliations:** 1 Biomedical Engineering Group, E.T.S. Ingenieros de Telecomunicación, Universidad de Valladolid, Campus Miguel Delibes, Paseo Belén 15, 47011 Valladolid, Spain; 2 Centro de Investigación Biomédica en Red de Bioingeniería, Biomateriales y Nanomedicina (CIBER-BBN), Madrid, Spain; 3 Servei d’Oftalmologia, Institut d’Investigació Sanitària Pere Virgili (IISPV), Hospital Universitari Sant Joan de Reus, Universitat Rovira i Virgili, Tarragona, Spain

**Keywords:** Lesion segmentation, Deep learning, Asymmetric dense U-Net, Retinal image analysis

## Abstract

Early detection of retinal lesions helps to avoid visual loss or blindness. The main lesions associated with eye diseases include soft exudates, hard exudates, microaneurysms, and hemorrhages. However, the segmentation of these four kinds of lesions is difficult and time-consuming due to their uncertainty in size, contrast, and high inter-class similarity. To address these issues, this study presents Deep Decoder-Focused U-Net (DDFU-Net), an asymmetric dense U-Net model for automatic and accurate multi-lesion segmentation using fundus images. Our approach simultaneously segments all four kinds of retinal lesions after proving that multi-task learning yields better results than single-task learning. DDFU-Net incorporates an asymmetric design with five dense blocks in the encoder and seven dense blocks in the decoder. This design enhances feature extraction while ensuring a more refined reconstruction of lesion boundaries, particularly for small and complex structures. By allocating more layers to the decoder, the model improves segmentation accuracy by gradually restoring spatial details lost during down-sampling, mitigating over-compression, and enhancing fine-grained feature preservation. Comprehensive experiments on IDRiD and DDR datasets well demonstrate the superiority of our approach, which outperforms state-of-the-art segmentation methods. Specifically, DDFU-Net achieved a mean Area Under the Precision-Recall Curve of 54.86%, a mean Intersection Over Union of 36.96%, and mean Dice scores of 52.24% on the DDR test set. On the IDRiD test set, it achieved 66.69%, 57.31%, and 69.93%, respectively. The asymmetric structure outperforms traditional symmetric U-Nets by capturing more detailed features during encoding while reducing complexity during decoding. The proposed method can be useful to aid in the diagnosis of eye diseases, reducing the workload of specialists and improving the attention to patients.

**Supplementary Information:**

The online version contains supplementary material available at https://doi.org/10.1007/s10439-026-04032-w.

## Introduction

Lesions such as microaneurysms (MAs), hemorrhages (HEs), hard exudates (EXs), and soft exudates (SEs) in fundus images are important manifestations for retinal fundus disease diagnosis by ophthalmologists [1, 2]. However, the examination of fundus images is time-consuming and small lesions are hard to observe [1, 3]. Therefore, to liberate ophthalmologists from heavy workload, automated lesion segmentation has become a trend [3] and many image processing and machine learning-based approaches for retinal lesion segmentation have been proposed [3–13].

Traditional retinal lesion segmentation approaches, based on mathematical morphology, thresholding, region growing, and pixel classification [4], have shown limited success due to the great morphological diversity of lesions and the similarity between lesions and other structures in fundus images [4–6]. These two problems have not been effectively solved until deep neural networks exploded in the field of computer vision [7]. Most existing segmentation methods focus on detecting one or two types of lesions at a time [8]. For instance, VRT, PATech, and iFLYTEK, which were the top three methods in the 2018 ISBI grand challenge ‘Diabetic Retinopathy - Segmentation and Grading’ [10], used a separate convolutional neural network (CNN) model for each lesion type, training four different patch-level CNN models for SEs, EXs, MAs, and HEs. However, the advantages of simultaneous multi-lesion segmentation have been recently recognized [3, 7, 9, 11, 12]. In this context, L-Seg incorporated side extraction layers into each convolutional layer group of a VGG network to extract and fuse features at different scales, coupled with a multi-channel bin loss function for simultaneous lesion segmentation [7]. Guo et al. [9] used an ensemble of fully CNNs with various input sizes to capture lesions at different scales. They further refined the segmentation by incorporating an additional CNN to fuse the outputs of these fully CNNs enhancing the overall segmentation accuracy. A novel network integrating self-attention and cross-attention blocks into U-Net for improved interaction between lesions and vessels was proposed in RTNet [3]. He et al. [11] addressed this by designing attention blocks to fuse feature maps from different depths, and Liu et al. [12] designed mechanisms to segment tiny lesions by reassembling features from multiple levels. A diabetic retinopathy detection framework that includes preprocessing steps like resizing, binarization, contrast enhancement, and morphological transformations, followed by simultaneous segmentation of MAs and HEs using an U-Net architecture was also proposed in [13].

Despite all the previous research, automated lesion segmentation remains challenging due to the following aspects:

1. Lesions in retinal fundus images exhibit a complex structure, characterized by significant variations in shape, size, and intensity across different regions [14]. These variations pose a substantial challenge for automated segmentation algorithms, as they must accurately capture the diverse morphological characteristics of lesions throughout the image. For instance, lesions can manifest as irregularly shaped structures with varying degrees of contrast and intensity, complicating the segmentation process [14].
2. The small size of many lesions, such as certain HEs, EXs, and MAs, which may only span a few pixels. The small size of these lesions makes them particularly challenging to differentiate from background noise and artifacts present in the image [14].
3. High inter-class similarity among different lesion types makes it challenging even for human experts to differentiate between them. Specifically, distinguishing between bright lesions, such as SEs and EXs, or between red lesions, such as HEs and MAs, remains a significant difficulty, despite the distinct visual differences between the two groups [7].
4. The presence of structures that mimic pathological lesions requires accurate differentiation. Such confounding structures include non-pathological anatomical features, such as reflective vessels and imaging artifacts, as well as distinct pathological lesions, such as drusen, which may be misidentified as EXs [7].

To address the above issues, we propose a Deep Decoder-Focused U-Net (DDFU-Net), a robust and accurate deep-learning model for segmentation of four retinal lesions, including MAs, HEs, EXs, and SEs. While asymmetric U-Nets and dense connections have been explored in foundational works [15], previous state-of-the-art architectures typically favor encoder-heavy designs, such as DeepLabv3+ [16] or Swin Transformer-based models [17], to maximize the extraction of abstract semantic features. Our study identifies a critical limitation in this standard approach for the specific domain of small-object segmentation. Excessive down-sampling in deep encoders leads to over-compression, a phenomenon where the fine-grained spatial details required to detect tiny lesions like MAs are lost [18]. These lesions often span only a few pixels making spatial preservation paramount [14]. Consequently, the DDFU-Net is designed to counter this issue by limiting the encoder depth to preserve spatial fidelity and extending the decoder depth. However, as this hypothesis regarding the superiority of decoder-focused patterns required verification, several ablation studies were performed to corroborate the proposed architectural adjustments. Furthermore, we performed a comparative evaluation between multi-task and single-task learning, confirming that the multi-task approach yields superior performance for this specific domain. The proposed model is tested on two public datasets, DDR [19] and IDRiD [20], to validate these architectural hypotheses. The novelty of this work lies in a four-fold contribution:

1. Proposal of an ablation-validated decoder-focused architecture. Unlike standard symmetric U-Nets or transfer-learning models that rely on heavy encoders [16, 17], we introduce a specific decoder-heavy asymmetry. We corroborate this design through ablation studies, proving that allocating more computational capacity to the up-sampling path solves the over-compression problem [18] and recovers micro-lesions that are otherwise lost.
2. Integration of dense connectivity for feature preservation. We integrate dense connectivity within this asymmetric structure to maximize the propagation of small spatial features. Our experiments confirm that this specific configuration handles the morphological diversity of lesions more effectively than standard connections.
3. Extensive comparative analysis between single-task lesion segmentation and multi-task lesion segmentation. We provide a comprehensive analysis quantifying the impact of multi-task learning on inter-class similarity. We demonstrate that our multi-task approach improves generalization and reduces false positives among confounding lesion types, such as distinguishing HEs from MAs, compared to isolated single-task models.
4. Comprehensive validation of the proposed model in multi-task lesion segmentation. Comparative assessments on the DDR and IDRiD datasets reveal that the DDFU-Net outperforms existing symmetric and ensemble-based segmentation models. This establishes DDFU-Net as a robust architectural solution to the trade-off between semantic abstraction and spatial precision in retinal imaging.

## Materials

Two datasets were used in this study, which aim to provide a wide range of image qualities and features. Thus, it allowed us an improved understanding of the performance of the architecture variants. The publicly available databases DDR [19] and IDRiD [20] were used in this work.

### DDR

DDR [19] dataset contains 757 color fundus images of size ranging from 1088×1920 to 3456×5184 pixels. Among these, 383 images are used for training, 149 for validation, and 225 for testing. In DDR, ophthalmologists have annotated 601, 570, 239, and 486 images as containing HEs, MAs, SEs, and EXs, respectively [19]. For experiments and comparison with other methods, we have used the training and validation sets for model training and validation, and we have evaluated the performance on the test set. Informed consent was obtained from all patients involved in this study [19].

### IDRiD

The IDRiD dataset [20] consists of 81 color fundus images sized at 4288×2848 pixels. Among these, 54 images are used for training and 27 for testing. Provided as part of the ‘Diabetic Retinopathy - Segmentation and Grading’ grand challenge in 2018, the IDRiD dataset includes 11716, 1903, 150, and 3505 regions annotated as EXs, HEs, SEs, and MAs, respectively [20]. To compare with other methods on IDRiD, we split the training set into 90% for training and 10% for validation. The training and validation subsets were used for model training and validation, while the test set was used for performance evaluation. Informed consent was obtained from all patients participating in this study [20].

## Methods

### Image Pre-processing

To address the variable image resolutions among the datasets, we conducted the following image preprocessing. First, we removed zero-pixel areas, which are black regions that do not contain information, by cropping around the inner circle of the retina [21]. Then, we resized the remaining image into a fixed image of 512×512 pixels [22]. Finally, we normalized the pixel values to the interval [0, 1] for a better training process [23].

### Data Augmentation

Data augmentation was adopted to reduce overfitting and increase the performance of the model [21]. In this work, the augmented images were obtained applying the following simple transformations: (i) scaling randomly by factor of [0.8, 1.2] with a step size of 0.1; (ii) rotating randomly within the range of [0, 360°] with a step size of 90°; (iii) adding gaussian noise to simulate the image quality variability present in fundus images [22, 24]. After data augmentation, the total number of training images in the DDR dataset were 12,768 and 1107 for the IDRiD dataset.

### Network Architecture

U-Net and its variants play an important role in medical image segmentation. However, the simple skip connections may ignore global features, focusing mainly on local details, such as edges and textures. This global context can be important for accurate segmentation [25]. Additionally, since skip connections pass detailed local information, they might also pass along irrelevant details, such as noise or minor local details that are not significant for the task, affecting network performance [25].

**Fig. 1:**
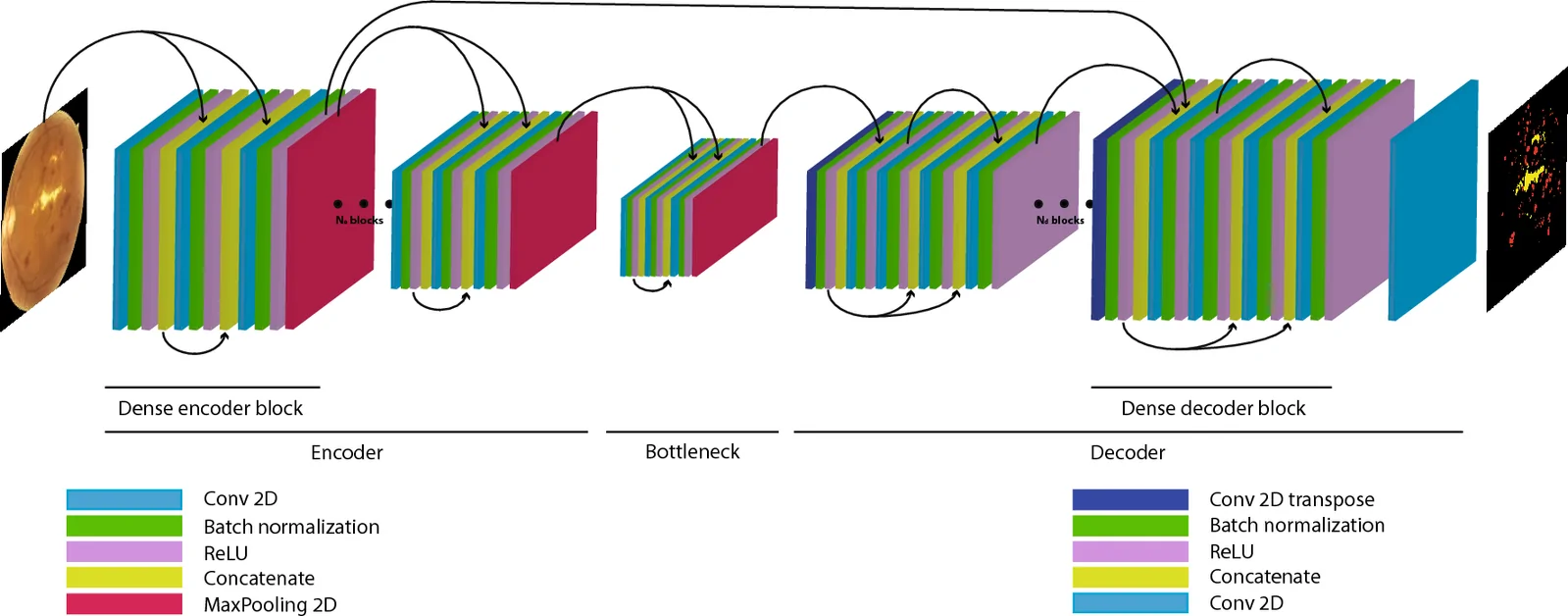
Design of the proposed dense U-Net architecture. $N_e$ blocks number of encoder blocks. $N_d$ blocks number of decoder blocks

This paper presents an enhanced asymmetric U-Net architecture, named DDFU-Net, which integrates dense blocks. Unlike the conventional U-Net, DDFU-Net uses asymmetric down-sampling and up-sampling paths to form an encoder–decoder structure. This innovative design reduces computational costs while preserving critical features, resulting in a model that is both fast and accurate. Another modification in DDFU-Net is the asymmetric expansion of the network, where the number of filters in each block of the encoder is doubled while maintaining the same number of filters in each block of the decoder [26, 27]. As detailed in Fig. 1 and Table 1, this design establishes a direct connectivity pattern where each layer receives inputs from all preceding layers within a block.

The proposed DDFU-Net constitutes a novel architectural framework that embeds dense connectivity throughout both the asymmetric down-sampling and up-sampling paths to maximize information flow. Dense blocks are incorporated at each stage of the U-Net, both in the down-sampling and up-sampling paths, ensuring direct connections between layers that facilitate efficient gradient propagation [28]. This design helps reduce the risk of the vanishing gradient problem by enabling information to flow through the network without significant attenuation, as each layer within a dense block receives inputs from preceding layers in the same block [28]. This mechanism enhances feature propagation and supports residual learning, reducing the risk of gradient degradation during backpropagation [28]. The architecture includes bottleneck layers of 1×1 convolutions within the dense blocks to improve computational efficiency by reducing the number of input feature maps [28]. This design leverages the benefits of dense connections to obtain the feature information from different receptive fields and residual learning, contributing to improve segmentation accuracy. Finally, to validate the specific advantages of our proposed asymmetric configuration, we have also developed a symmetric dense U-Net architecture, characterized by identical encoder and decoder depths for comparative evaluation. However, due to hardware memory constraints observed when scaling the symmetric model beyond seven dense blocks, we restricted the maximum depth of the asymmetric configuration to seven blocks to ensure experimental consistency.

**Table 1:** Detailed architectural specifications of DDFU-Net blocks

Block type	Layer operation	Kernel size	Stride/pool	Filters	Input source
Encoder block	Conv2D + BN + ReLU	3×3	1	128	Block Input
	Conv2D + BN + ReLU	3×3	1	128	Concat (input, previous)
	Bottleneck Conv2D + BN + ReLU	1×1	1	128	Concat (input, all previous)
	Max pooling	2×2	2×2	–	Bottleneck output
Decoder block	Transpose Conv2D + BN + ReLU	3×3	2×2	64	Previous block output
	Conv2D + BN + ReLU	1×1	1	64	Concat (up-sampled, skip connection)
	Conv2D + BN + ReLU	3×3	1	64	Concat (previous, input)
	Bottleneck Conv2D + BN + ReLU	1×1	1	64	Concat (all previous)

*BN* batch normalization, *ReLU* rectified linear unit

#### Encoder Block

The down-sampling path of DDFU-Net enhances the traditional U-Net architecture by incorporating densely connected convolutional layers. Unlike the plain feed-forward model of U-Net, DDFU-Net features a dense structure where each layer receives input from all preceding layers, improving feature propagation [28].

As summarized in Table 1, the encoder path includes $N_e$ dense blocks, each comprising multiple convolutional layers, batch normalization layers, ReLU activation functions, and concatenation operations. The first convolutional layer in each dense block uses a 3 × 3 kernel, and we ensured that the output size matches the input size. After the convolution, batch normalization is applied, followed by the ReLU activation function. The second convolutional layer also uses a 3 × 3 kernel, with its input being the concatenation of the block input and the output from the first convolutional layer. This layer is also followed by batch normalization and ReLU activation [28]. The third convolutional layer uses a 1 × 1 kernel and takes the concatenation of the original input and the outputs from the first two convolutional layers as its input. It also undergoes batch normalization and ReLU activation. Finally, a max pooling layer with a pool size of 2 × 2 is applied to down-sample the output, reducing its spatial dimensions by half [28]. The number of filters in each convolutional layer is the same for each block, which is 128.

#### Decoder Block

In the decoder part of DDFU-Net, we implement an up-sampling mechanism designed to effectively reconstruct high-resolution feature maps from the encoded representations, as presented in Fig. 1. This decoder path includes $N_d$ identical blocks which specific parameters are listed in Table 1.

The up-sampling block, shown in Fig. 1, begins by taking two inputs: (i) the output from the previous layer in the decoder path, and (ii) the corresponding feature map from the encoder path. The first step involves applying a transpose convolution with a kernel size of 3×3 and strides of 2×2. This operation up-samples the input feature map, effectively doubling its spatial dimensions. Batch normalization and a ReLU activation function are then applied to stabilize the training process and introduce non-linearity [28]. Crucially, the skip connection from the encoder is then concatenated directly with this up-sampled feature map. This concatenated feature map undergoes a 1×1 convolution, followed by batch normalization and ReLU activation. This first convolutional step serves to refine the combined feature map by learning a weighted combination of the input channels. Further refinement is achieved through dense connections. The output from the previous step is concatenated with the initial inputs and processed by a 3×3 convolution, followed by batch normalization and ReLU activation. To fully leverage the dense architecture, the final step in the block involves concatenating the outputs of all preceding layers and applying a final 1×1 convolution. This produces the final block output while reducing the channel depth for computational efficiency. Batch normalization and ReLU activation are applied once more to ensure the output feature map is well-normalized [28]. The number of filters in each convolutional layer is the same for each block, which is 64 [26, 27].

### Analysis of Dense Connectivity

To address the theoretical basis for utilizing dense connections in small lesion segmentation, we formalize the dense block as a preserver of high-frequency spatial components [29].

Let $x_l$ denote the output of the *l*-th layer within a dense block. Unlike standard convolutional chains where $x_l = H_l(x_{l-1})$, the *l*-th layer receives the feature maps of all preceding layers as input [29]:

1 $$\begin{aligned} x_l = H_l([x_0, x_1, \dots , x_{l-1}]), \end{aligned}$$

where [⋯] represents the channel-wise concatenation operator and $H_l(\cdot )$ is a composite function of batch normalization, ReLU, and convolution.

The rationale for this architecture is driven by the nature of retinal lesions which often occupy only a few pixels and represent high-frequency spatial variations $\xi _{HF}$ [29]. In a standard feed-forward network, repeated application of pooling operations $\mathcal {P}(\cdot )$ acts as a low-pass filter, causing the attenuation of these fine-grained details:

2 $$\begin{aligned} \lim _{L \rightarrow \infty } \mathcal {P}^L(x_0) \approx \bar{x}_{low\_freq}. \end{aligned}$$

By employing the concatenation mechanism defined in Eq. (1), the network ensures that the initial high-frequency components $x_0$ remain explicitly accessible at deeper layers $x_l$. The final transition layer of the block processes a composite feature map $\textbf{X}_{out} = [x_0, \dots , x_L]$. This allows the decision boundary to be formed using both the abstract, semantic features derived from $x_L$ and the raw, high-frequency edge information preserved in $x_0$ [29].

Furthermore, this architecture implicitly modulates the Effective Receptive Field (ERF). While $x_0$ possesses a small, local receptive field focused on texture, subsequent layers $x_l$ possess increasingly larger receptive fields capturing global context. The dense concatenation implies that the effective receptive field at the block output is a weighted superposition of multiple scales [29]:

3 $$\begin{aligned} ERF_{total} \propto \sum _{i=0}^{L} w_i \cdot ERF(x_i), \end{aligned}$$

where $w_i$ represents the learnable weights of the final 1×1 convolutional transition layer, determining the contribution of the receptive field of the *i*-th layer to the total effective receptive field.

### Asymmetric Variants

**Fig. 2:**
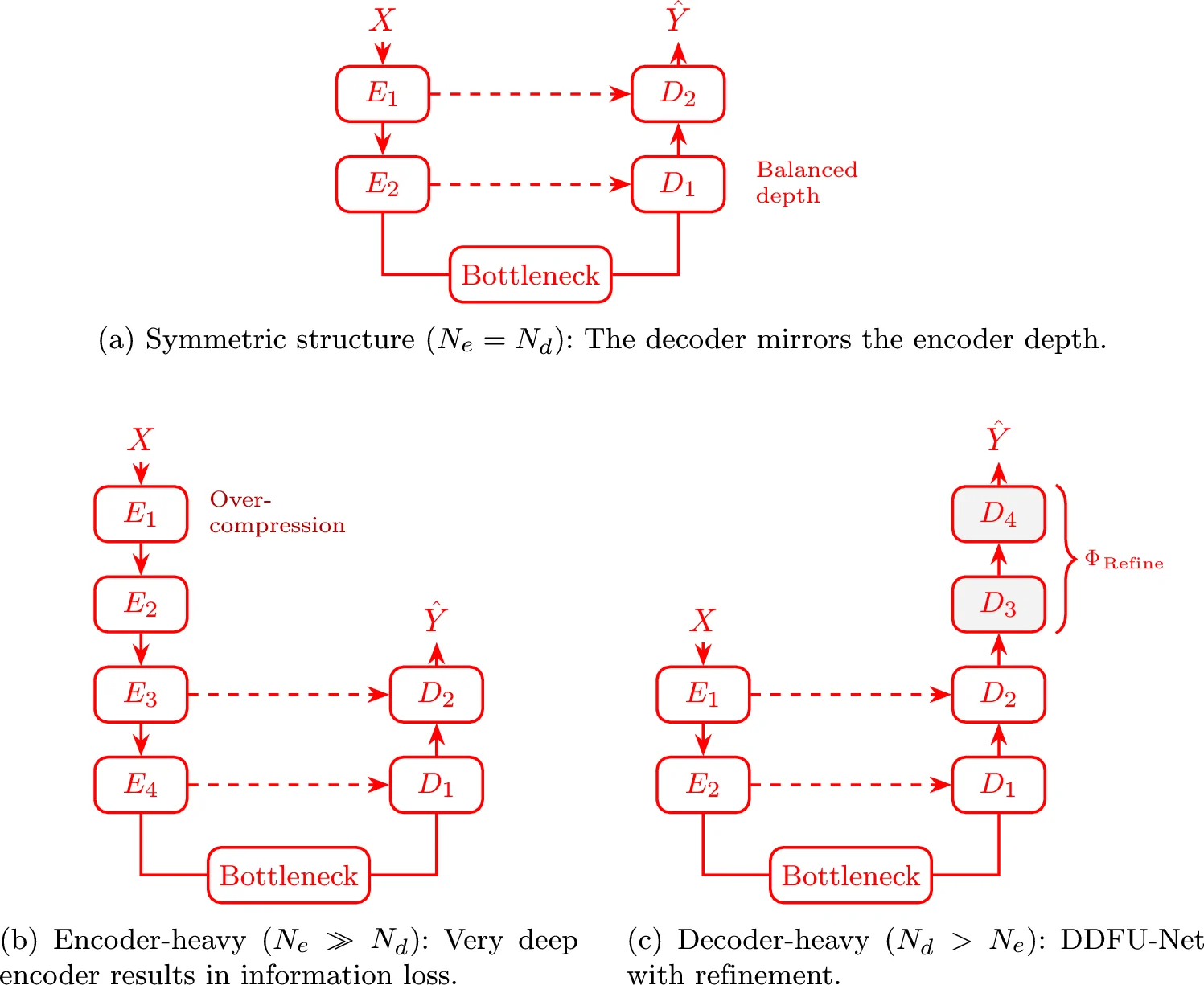
Structural comparison of U-Net variants. **a** Symmetric architecture. **b** Encoder-heavy architecture with increased depth. **c** Decoder-heavy architecture (DDFU-Net)

To provide a formal justification for the proposed asymmetric design, we model the DDFU-Net as a composition of non-linear feature transformation functions mapping $\mathcal {F}: \mathcal {X} \rightarrow \mathcal {Y}$ [30]. Let *E*(*X*) denote the encoder operator comprising a sequence of $N_e$ dense down-sampling blocks. We define the relationship between encoder and decoder depths to analyze three structural variants shown in Fig. 2.

In the standard symmetric architecture (Fig. 2a), the decoder depth $N_d$ equals the encoder depth $N_e$ ($N_d = N_e$). The network functions as a mirrored auto-encoder where the objective is the approximation of the inverse encoder mapping to recover spatial resolution [31]. The output $\hat{Y}_{sym}$ is defined by a direct layer-wise inversion:

4 $$\begin{aligned} \hat{Y}_{sym} = D_{N_e} \left( D_{N_e-1} \left( \dots D_1(E(X)) \right) \right), \end{aligned}$$

here, every up-sampling block $D_i$ is structurally paired with a corresponding encoder block via skip connections. The mapping is constrained to $\mathcal {Z} \rightarrow \mathcal {Y}$, focusing solely on up-scaling low-resolution features back to the input grid size.

In the encoder-heavy variant (Fig. 2b), the encoder is deeper than the decoder ($N_e> N_d$). Mathematically, this creates an information bottleneck. The deep encoder transforms the input into a highly abstract, low-resolution latent space $\mathcal {Z}_{deep} = E_{N_e}(\dots E_1(X))$. The shallow decoder, having fewer stages ($N_d < N_e$), acts as a truncated inverse operator:

5 $$\begin{aligned} \hat{Y}_{enc} \approx D_{N_d} \left( \dots D_1(\mathcal {Z}_{deep}) \right). \end{aligned}$$

Increasing the depth of the encoder drastically increases the effective receptive field, often at the cost of high-frequency spatial information retention [29]. Because the decoder lacks the corresponding number of up-sampling steps to match down-sampling operations of the encoder, the high-frequency spatial information lost during deep compression cannot be fully recovered. This could lead to the over-compression phenomenon highlighted in Fig. 2b, where small lesions (fine spatial details) are attenuated.

In the proposed DDFU-Net (Fig. 2c), where $N_d> N_e$, we formulate the asymmetric decoder as a composite function of reconstruction and refinement operators. The output $\hat{Y}$ is defined by the recursive composition:

6 $$\begin{aligned} \hat{Y} = D_{N_d} \left( D_{N_d-1} \left( \dots D_1(E(X)) \right) \right) \end{aligned}$$

To clarify the functional difference between symmetric and asymmetric architectures, we divide this composition chain at index $N_e$ (the depth of the encoder). This allows us to decompose Eq. 6 into two distinct operational phases:

7 $$\begin{aligned} \hat{Y} = \underbrace{D_{N_d} \bigg ( \dots D_{N_e+1}}_{\Phi _{\text {Refine}}} \bigg ( \underbrace{D_{N_e} \Big ( \dots D_1 (E(X)) \Big )}_{\text {Reconstruction}} \bigg ) \dots \bigg ) \end{aligned}$$

1. **Symmetric reconstruction** ($D_1 \dots D_{N_e}$): The first $N_e$ blocks mirror the encoder. Their mathematical role is to invert the pooling operators via transposed convolutions, recovering the feature map to the original input resolution $V_0$.
2. **Non-linear refinement** ($\Phi _{\text {Refine}}$): The additional blocks $D_{N_e+1}$ through $D_{N_d}$ (highlighted in Fig. 2c) constitute a refinement operator $\Phi _{\text {Refine}}$ applied at the full spatial resolution. Unlike the preceding layers, these blocks do not perform scaling. Instead, they act as a discrete dynamical system:
  8 $$\begin{aligned} h_{k+1} = h_k + f(h_k), \quad \text {for } k = N_e \dots N_d, \end{aligned}$$
  where *f* represents the dense block residuals.

We define the encoder *E* as a lossy down-sampling operator $\mathcal {P}: \mathcal {X} \rightarrow \mathcal {Z}$. This operator effectively acts as a low-pass filter, suppressing high-frequency spatial details crucial for delineating small lesions. The goal of the decoder $D_\theta$ is to learn an approximate inverse $\mathcal {P}^{-1}$ that minimizes the expected loss:

9 $$\begin{aligned} \min _\theta \mathbb {E} \left[ \mathcal {L} \left( Y, D_\theta (\mathcal {P}(X)) \right) \right], \end{aligned}$$

where $\mathcal {L}$ is the segmentation loss function. As noted in the structural decomposition, by increasing the decoder depth ($N_d> N_e$), we increase the functional capacity of $D_\theta$ to approximate $\mathcal {P}^{-1}$. The additional non-linear refinement operators $\Phi _{\text {Refine}}$ allow the model to iteratively minimize the error in Eq. 9, specifically targeting the high-frequency boundaries that are typically attenuated by the lossy operator $\mathcal {P}$.

Based on this formulation, we propose two architecture variants (Fig. 2) with modified strides to achieve different levels of down-sampling and up-sampling:

- **Increased depth in the encoder relative to the decoder** ($N_e> N_d$): Based on convolutional network design principles and U-Net-based segmentation approaches [15, 28], increasing the encoder depth could enhance feature extraction capabilities. This design focuses computational resources on $\mathcal {E}$, which can be beneficial for tasks that involve detecting complex patterns or high-level semantic features. However, for small-object segmentation, this risks excessive spatial compression that a shallow decoder cannot recover.
- **Increased depth in the decoder relative to the encoder** ($N_d> N_e$): As formalized in Eq. 6 and illustrated in Fig. 2c, increasing the depth of the decoder enhances the ability of the model to reconstruct high-resolution outputs. By having more blocks in the decoder, the model applies more refinement operators $\Phi _{\text {Refine}}$ to the feature maps. This ensures that detailed structures are accurately reconstructed from lower-resolution feature representations obtained during down-sampling. Additionally, the increased decoder depth helps in better handling complex textures and boundaries in segmentation tasks.

These architecture variants offer enhanced flexibility in handling different down-sampling and up-sampling requirements, ensuring that DDFU-Net can be effectively applied to a wide range of image segmentation tasks.

### Experimental Learning Framework

To evaluate the efficacy of joint lesion segmentation, we implemented two different learning frameworks: a single-task learning baseline and a multi-task learning approach.

#### Single-Task Learning

In the single-task setup, we treat the segmentation of each lesion type as an independent problem [32]. This involves instantiating and training four separate models, one for each specific lesion class (MA, HE, EX, and SE). Each model has its own independent encoder and decoder weights and is trained to output a binary probability map for its designated target. While this allows each model to specialize exclusively on one lesion type, it ignores the potential correlation between different lesions and significantly increases the computational resources required for training and inference [32].

#### Multi-Task Learning

We frame the segmentation as a multi-task learning problem implemented via hard parameter sharing [33]. Instead of separate networks, we utilize a single model with a shared encoder and decoder backbone. The network branches only at the final output layer to simultaneously produce a 5-channel probability map (background, EX, SE, HE, MA). We hypothesize that this joint training approach enhances performance through two primary mechanisms:

- **Shared feature representation**: Different lesion types often share underlying spatial dependencies with retinal structures, such as proximity to blood vessels. By sharing the encoder weights, the model learns a more robust global representation of the fundus image that benefits the segmentation of all lesion classes [33].
- **Regularization effect**: Training on multiple tasks simultaneously is expected to act as a regularizer, reducing the risk of overfitting to the specific details or noise of a single lesion type [33]. The auxiliary tasks of segmenting other lesions provide additional information that help the encoder learn more generalized features, which is particularly beneficial for classes with fewer training samples, such as SEs.

### Training Details

In the training stage, we used the cross-entropy (CCE) loss as the objective function. We selected CCE because it is a fundamental distribution-based loss that minimizes the Kullback–Leibler divergence between the predicted and ground-truth distributions [34]. Unlike region-based losses, which can suffer from training instability or vanishing gradients when predictions and targets do not overlap, CCE provides a smooth optimization landscape that stabilizes the convergence of the network during the early training epochs [34]. The CCE is defined as follows [11]:

10 $$\begin{aligned} L_{ce}(y,g)=-\sum _{i=1}^{k}{g_{i} \cdot log(y_{i})}, \end{aligned}$$

where *g*, *y*, and *k* are the ground truth, predicted lesion maps of the input fundus images, and the number of lesion types, respectively [11].

For the multi-task framework, we hypothesize that the joint training of multiple lesion classes mitigates the impact of class imbalance through the regularization effect of concurrent segmentation tasks [33], allowing the model to leverage shared features without requiring complex loss weighting schemes. For the single-task framework, we maintained the same CCE objective to ensure a fair, direct comparison of architectural efficacy.

All experiments were conducted on a workstation equipped with an NVIDIA RTX 4080 GPU to ensure computational efficiency. A batch size of 2 was selected to mitigate GPU memory constraints, while preserving the capability to process high-resolution feature maps necessary for lesion detection. The network parameters were optimized using the Adam optimizer with an initial learning rate of $1 \times 10^{-4}$. To enhance training efficiency and minimize overfitting, we implemented an adaptive learning rate scheduling strategy, where the learning rate was reduced by a factor of 0.1 if the validation loss failed to improve for 3 consecutive epochs and a minimum lower bound of $1 \times 10^{-9}$. Additionally, we used an early stopping mechanism with a patience of 10 epochs to automatically finish training when convergence was reached and retain the best-performing model weights. The DDR dataset was utilized to find the optimal architecture, which was subsequently applied to training and testing on both the DDR and IDRiD datasets to evaluate the performance of the proposed model.

### Evaluation Metrics

To assess the performance of the proposed architecture variants, we employed several widely recognized metrics used in fundus image segmentation research. These metrics include class-wise Dice coefficient [35], mean class-wise Dice (mDice), class-wise Intersection Over Union (IoU) [36], mean class-wise IoU (mIoU), class-wise Area Under the Precision-Recall Curve (AUPR) [37], and mean class-wise AUPR (mAUPR).

The Precision, Recall, and AUPR metrics are defined as follows [37, 38]:

11 $$\begin{aligned} Precision = \frac{TP}{TP + FP} \end{aligned}$$

12 $$\begin{aligned} Recall = \frac{TP}{TP + FN} \end{aligned}$$

13 $$\begin{aligned} AUPR = \sum _{n=1}^{N} (R_{n} - R_{n-1}) \times P_{n}, \end{aligned}$$

where *TP*, *FP*, and *FN* stand for true positive, false positive, and false negative, respectively; $R_{n}$ and $P_{n}$ are the Recall and Precision scores, respectively, at the $n_{th}$ threshold, and *N* is the number of thresholds. The AUPR can be computed based on several pairs of Precision and Recall values so that it is not affected by *TP*. Consequently, the AUPR metric is not affected by the data imbalance presented in the datasets used in this study. On the other hand, Dice and IoU metrics are defined as follows [35, 36]:

14 $$\begin{aligned} Dice = \frac{2 \times TP}{2 \times TP + FP + FN} \end{aligned}$$

15 $$\begin{aligned} IoU = \frac{TP}{TP + FP + FN} \end{aligned}$$

## Results

In this section, we present ablation studies to show the impact of the multi-task learning and network structure. We conducted experiments using the DDR dataset, chosen for its extensive number of annotated images. The DDR dataset is particularly suitable for ablation studies because it provides high-quality annotations for multiple lesion types. To demonstrate the effectiveness of asymmetric structures, we performed comparative experiments with various symmetric and asymmetric combinations of encoder–decoder architectures. Additionally, we conducted experiments to validate whether multi-task lesion segmentation demonstrates advantages over single-task lesion segmentation. After optimizing the hyperparameters using DDR, the final configuration was also trained and evaluated on the IDRiD database.

**Table 2:** Segmentation results with different symmetric network structures for single-task lesion segmentation

Number of down-sampling blocks	Number of up-sampling blocks	Number of parameters	AUPR					IoU					Dice				
			mAUPR	EX	HE	SE	MA	mIoU	EX	HE	SE	MA	mDice	EX	HE	SE	MA
2	2	1,342,785	46.84	59.66	47.13	45.28	35.30	20.75	37.90	21.49	12.89	10.71	32.42	53.29	33.33	25.75	17.30
3	3	5,080,001	50.07	59.79	48.38	*53.73*	**38**.**36**	29.65	37.14	23.57	37.29	**20**.**58**	44.18	52.90	38.15	54.32	**31**.**36**
4	4	19,635,777	47.42	60.40	*52.62*	45.28	31.38	27.83	38.35	24.10	32.29	16.58	40.39	53.75	38.84	41.61	27.36
5	5	77,258,945	*51.72*	*60.83*	**54**.**35**	53.38	*38.31*	*32.21*	**40**.**09**	**29**.**34**	*40.05*	*19.35*	**47**.**36**	**55**.**78**	**45**.**37**	*57.20*	*31.07*
6	6	306,737,985	**51**.**95**	**60**.**88**	52.25	**58**.**81**	35.86	**32**.**30**	*39.54*	*29.23*	**41**.**61**	18.81	*46.85*	*54.80*	*43.66*	**58**.**77**	30.17
7	7	1,222,813,121	49.84	60.66	52.07	51.03	35.60	30.92	37.21	29.13	38.12	19.23	45.57	54.24	41.81	55.20	31.03

Results marked in bold are the highest ones while those in italic are the second highest ones

**Table 3:** Segmentation results with different symmetric network structures for multi-task lesion segmentation

Number of down-sampling blocks	Number of up-sampling blocks	Number of parameters	AUPR					IoU					Dice				
			mAUPR	EX	HE	SE	MA	mIoU	EX	HE	SE	MA	mDice	EX	HE	SE	MA
2	2	1,342,882	45.83	56.39	43.76	46.91	36.27	26.30	32.81	32.80	25.91	13.67	39.05	46.60	46.59	41.15	21.85
3	3	5,080,098	47.84	57.01	49.60	50.21	34.54	28.37	33.77	33.76	33.03	12.93	41.28	47.50	47.49	49.65	20.49
4	4	19,635,874	50.78	**61**.**39**	*55.15*	50.67	35.92	33.84	40.26	**41**.**25**	37.65	16.18	47.44	*54.90*	*54.92*	54.70	25.23
5	5	77,259,042	*51.48*	60.72	**55**.**54**	*54.28*	35.37	**35**.**16**	**40**.**71**	*40.47*	41.09	**18**.**35**	**49**.**62**	**55**.**14**	**55**.**06**	58.24	**30**.**03**
6	6	306,738,082	**52**.**09**	*60.86*	53.98	**55**.**80**	**37**.**71**	*34.93*	*40.46*	38.48	**43**.**59**	*17.19*	*49.00*	54.86	53.74	**60**.**71**	*26.72*
7	7	1,222,813,218	51.47	59.95	54.76	54.06	*37.11*	32.20	40.37	30.43	*41.20*	16.78	47.05	54.77	46.66	*60.13*	26.65

Results marked in bold are the highest ones while those in italic are the second highest ones

### Ablation Study on Different Symmetric Network Structures

We first evaluate the effect of the number of down-sampling and up-sampling blocks in the encoder and decoder dense U-Net with a symmetric structure. Tables 2 and 3 show results for single-task lesion segmentation and multi-task lesion segmentation, respectively. From Tables 2 and 3, dense U-Net with five down-sampling and up-sampling blocks yielded the highest average performance in terms of IoU and Dice.

As the network depth increases, improvements are observed in both single-task and multi-task lesion segmentation. However, when the network comprises more than 5 dense blocks in the encoder and decoder, results get worse. The most probable reason is that small lesions reside in only a few slices and more down-sampling layers are more likely to miss features for the small lesions in the deeper layers.

### Ablation Study on Different Asymmetric Network Structures

In the traditional U-Net, the decoder is symmetric with the encoder [15]. However, exploring asymmetric encoder–decoder configurations can optimize network efficiency and performance. To assess the effect of different encoder and decoder depths, we evaluated the segmentation performance of a dense U-Net with varying numbers of down-sampling layers in the encoder and up-sampling layers in the decoder using Dice, IoU, and AUPR metrics. Tables 4 and 5 show results for single-task lesion segmentation and multi-task lesion segmentation, respectively.

**Table 4:** Segmentation results with different asymmetric network structures for single lesion segmentation

Number of down-sampling blocks	Number of up-sampling blocks	Number of parameters	AUPR					IoU					Dice				
			mAUPR	EX	HE	SE	MA	mIoU	EX	HE	SE	MA	mDice	EX	HE	SE	MA
2	4	1,497,905	50.47	62.02	49.56	48.71	41.58	29.93	**42**.**39**	25.28	31.00	21.04	42.90	56.66	40.35	39.84	**34**.**76**
2	5	1,565,145	51.94	61.71	50.10	53.88	40.57	30.71	*41.41*	26.17	33.39	20.30	44.90	56.28	39.33	52.19	32.67
2	6	1,629,805	52.98	63.17	50.46	54.82	*43.48*	30.46	40.68	23.05	36.60	21.49	44.51	*57.83*	34.06	53.59	32.54
2	7	1,693,175	53.79	*63.22*	50.56	55.77	43.40	30.64	40.80	25.34	34.47	21.30	44.70	57.81	36.48	48.93	31.92
3	5	5,235,121	52.06	61.66	50.17	55.26	40.02	30.87	38.74	28.55	33.77	21.06	46.50	55.63	39.79	56.44	33.62
3	6	5,302,361	54.06	62.37	50.56	*60.83*	42.48	30.88	37.92	29.87	32.93	**22**.**80**	*47.32*	56.24	38.82	59.82	*34.44*
3	7	5,367,021	*54.19*	62.96	50.65	58.18	42.62	30.89	38.96	28.78	33.98	*22.46*	45.59	57.18	38.63	50.85	33.96
4	2	19,381,633	52.92	56.14	*53.72*	**61**.**38**	40.43	**33**.**30**	35.67	**32**.**74**	**43**.**57**	21.20	**49**.**01**	52.58	**49**.**33**	**60**.**70**	32.41
4	6	19,790,897	53.45	61.58	52.66	59.64	37.77	30.87	38.33	28.98	34.36	20.16	46.92	55.68	40.02	58.31	31.10
4	7	19,858,137	53.92	62.51	52.65	58.21	39.63	30.79	38.86	28.82	34.03	20.74	45.46	56.70	39.91	50.16	31.79
5	2	76,769,409	48.68	56.42	46.44	53.72	38.11	27.48	35.00	26.15	31.25	18.84	40.82	51.03	38.69	45.43	29.76
5	3	77,004,801	48.37	56.88	46.31	52.95	36.54	28.14	36.04	24.71	33.54	18.74	41.60	52.01	36.02	47.65	29.87
5	7	77,414,065	**55**.**27**	**63**.**95**	**54**.**30**	58.43	**44**.**40**	31.98	41.31	28.76	35.76	22.08	45.37	**58**.**47**	44.67	45.13	33.20
6	2	305,839,745	39.31	52.49	31.21	36.40	37.12	16.72	28.07	14.52	8.62	15.66	25.43	41.32	18.65	15.86	25.87
6	3	306,248,449	38.84	53.68	31.09	35.93	34.67	19.48	32.62	13.05	17.09	15.17	29.94	47.03	17.18	29.19	26.35
6	4	306,483,841	44.63	58.82	48.12	38.97	35.59	26.89	37.70	20.63	30.12	16.57	36.89	51.59	37.01	32.73	27.81
7	2	1,221,159,553	41.17	53.82	34.97	38.80	37.08	19.26	30.40	17.11	15.39	16.02	28.81	44.10	25.13	24.45	26.24
7	3	1,221,914,881	41.29	54.72	35.84	38.11	36.18	21.00	33.42	16.14	21.67	15.82	31.73	47.65	23.80	30.70	26.83
7	5	1,222,558,977	51.89	57.99	52.87	57.94	38.76	*32.36*	36.62	*31.25*	*43.06*	18.52	46.93	50.78	*47.61*	*60.20*	29.12

Results marked in bold are the highest ones while those in italic are the second highest ones

**Table 5:** Segmentation results with different asymmetric network structures for multi-task lesion segmentation

Number of down-sampling blocks	Number of up-sampling blocks	Number of parameters	AUPR					IoU					Dice				
			mAUPR	EX	HE	SE	MA	mIoU	EX	HE	SE	MA	mDice	EX	HE	SE	MA
2	4	1,498,002	53.28	63.39	52.76	53.62	*43.36*	32.59	41.99	29.38	39.61	19.38	47.43	58.02	42.46	56.74	32.48
2	5	1,565,242	53.32	63.48	52.93	53.44	**43.43**	33.10	42.52	29.78	39.73	20.37	47.80	58.42	42.98	56.93	32.87
2	6	1,629,902	53.25	63.94	53.60	52.68	42.79	34.34	44.45	30.45	40.06	**22**.**41**	48.60	59.06	44.19	57.21	33.94
2	7	1,693,272	53.35	*64.12*	53.79	52.78	42.71	32.12	*44.83*	30.68	40.21	12.76	48.90	59.31	44.53	57.42	34.34
3	5	5,235,218	52.83	63.21	53.99	52.11	41.99	34.20	42.53	31.02	42.48	20.77	49.50	58.41	46.52	59.48	33.59
3	6	5,302,458	53.19	63.49	54.36	52.47	42.43	34.73	42.91	31.65	43.18	21.17	50.21	58.70	47.26	60.32	**34**.**55**
3	7	5,367,118	53.52	63.82	54.79	53.07	42.38	35.13	43.51	32.19	43.83	20.99	50.55	59.21	47.98	61.02	33.99
4	2	19,381,730	52.36	61.33	54.19	*56.47*	37.43	36.59	41.81	**41**.**78**	44.14	18.64	51.37	58.97	**56**.**58**	61.24	28.68
4	6	19,790,994	53.80	63.53	55.49	55.97	40.21	36.70	43.82	*37.51*	45.49	19.98	51.70	59.81	*53.51*	62.49	30.99
4	7	19,858,234	*54.20*	*64.11*	*55.99*	55.79	40.91	*36.80*	44.51	35.51	46.00	21.19	*51.98*	*60.21*	51.91	62.99	32.79
5	2	76,769,506	43.00	55.03	42.51	38.99	35.47	28.00	35.03	32.99	26.99	16.99	40.05	50.07	47.99	38.99	23.14
5	3	77,004,898	48.00	59.03	48.99	47.02	36.97	32.05	39.52	32.99	36.21	19.48	46.00	55.02	49.01	50.99	28.98
5	7	77,414,162	**54**.**86**	**64**.**71**	**56**.**56**	55.67	42.49	**36**.**96**	**45**.**15**	33.93	**46**.**73**	*22.01*	**52**.**24**	**60**.**42**	50.66	**63**.**69**	*34.20*
6	2	305,839,842	37.10	50.29	36.56	29.56	31.98	18.16	26.55	26.53	8.63	10.92	28.62	41.18	39.49	15.88	17.93
6	3	306,248,546	38.79	54.23	35.53	33.47	31.92	23.31	32.91	32.59	14.17	13.55	35.87	47.40	47.39	24.82	23.86
6	4	306,483,938	45.00	56.51	45.01	44.99	33.49	29.01	37.03	33.51	29.99	15.49	43.00	52.99	48.99	44.01	25.99
7	2	1,221,159,650	33.75	46.48	33.97	26.98	27.57	15.00	24.03	23.99	5.99	6.01	26.00	39.01	36.99	12.01	15.99
7	3	1,221,914,978	40.50	50.04	38.03	37.02	36.91	20.50	29.02	27.51	13.51	11.96	31.50	44.02	40.98	22.52	18.48
7	5	1,222,559,074	53.03	58.58	*55.76*	**63**.**48**	34.30	34.99	41.44	35.07	*46.34*	17.10	50.28	58.60	51.25	*63.33*	27.93

Results marked in bold are the highest ones while those in italic are the second highest ones

### Ablation Study on Single-Task vs. Multi-Task Lesion Segmentation

We have also investigated the effect of multi-task learning in our work. We make a comparison between single-task learning and multi-task learning, and the results of them are listed in Tables 2 and 4 for single-task learning, and Tables 3 and 5 for multi-task learning. The results of multi-task learning are slightly higher than those of single-task learning in all indicators. One of the possible reasons is that learning together with other lesions can increase the ratio of foreground pixels, which means that the network can learn more information and get a full understanding of the lesion and retinal anatomic structure.

### Optimum Architecture

This study explores the training and evaluation of a dense U-Net model to identify the optimal combination of encoder and decoder blocks. Additionally, the performance of single-task learning was compared to multi-task learning to determine the most effective approach for retinal lesion segmentation. As shown in Tables 4 and 5, the highest results for both single-task lesion segmentation and multi-task lesion segmentation were achieved using an asymmetric dense U-Net configuration with five down-sampling blocks and seven up-sampling blocks. Additionally, the results indicate that multi-task lesion segmentation outperforms single-task segmentation, demonstrating the advantages of leveraging shared features across lesion types.

**Fig. 3:**
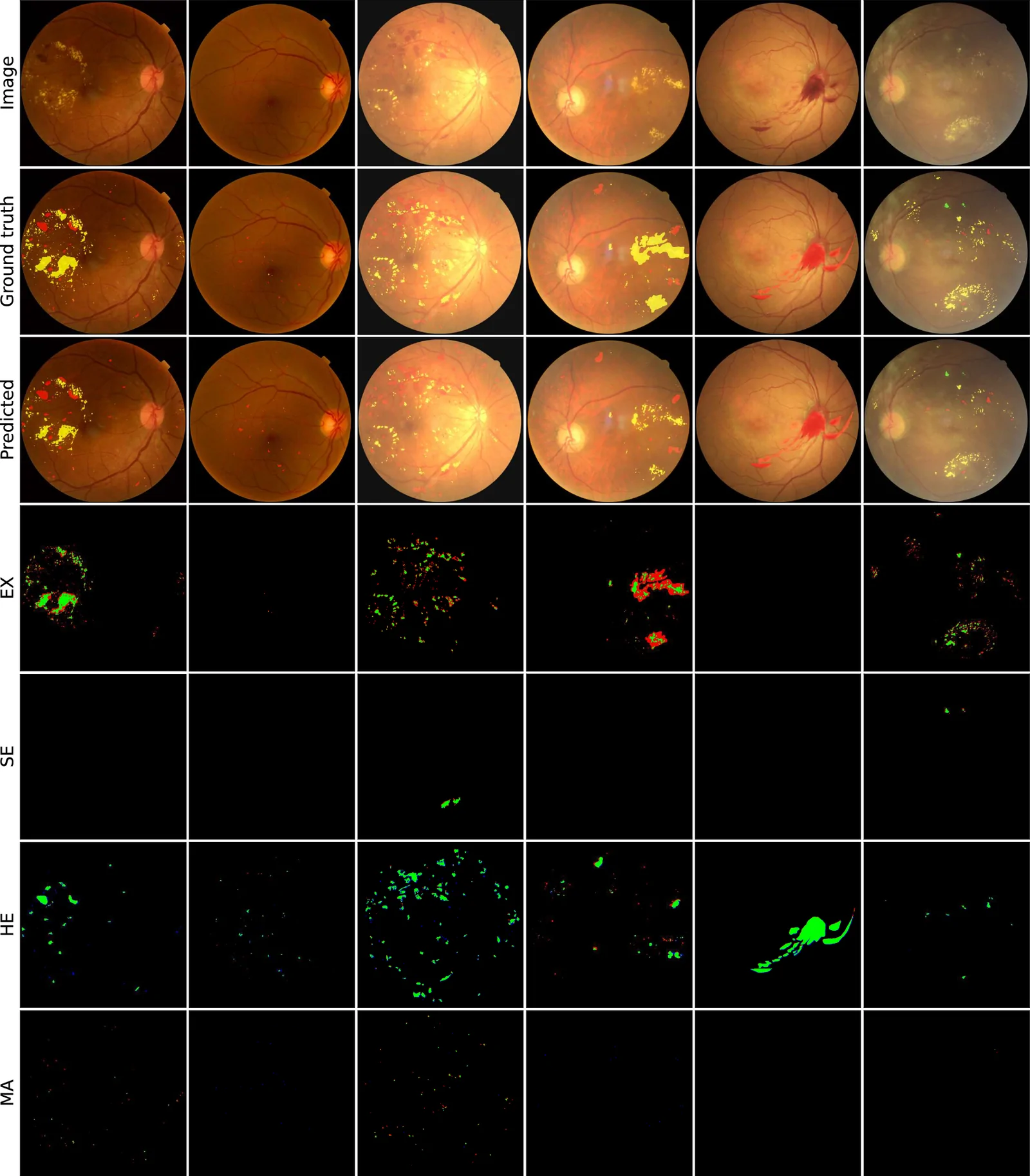
Segmentation results on the DDR test set. The first row shows the original images. The second and third rows present the ground truth and model predictions, respectively, using a lesion-specific color map: yellow (EX), green (SE), red (HE), and orange (MA). Rows 4 through 7 display the results for each lesion type individually. In these rows, the color coding reflects performance: green pixels represent true positives, red pixels indicate false negatives, and blue pixels denote false positives.

**Fig. 4:**
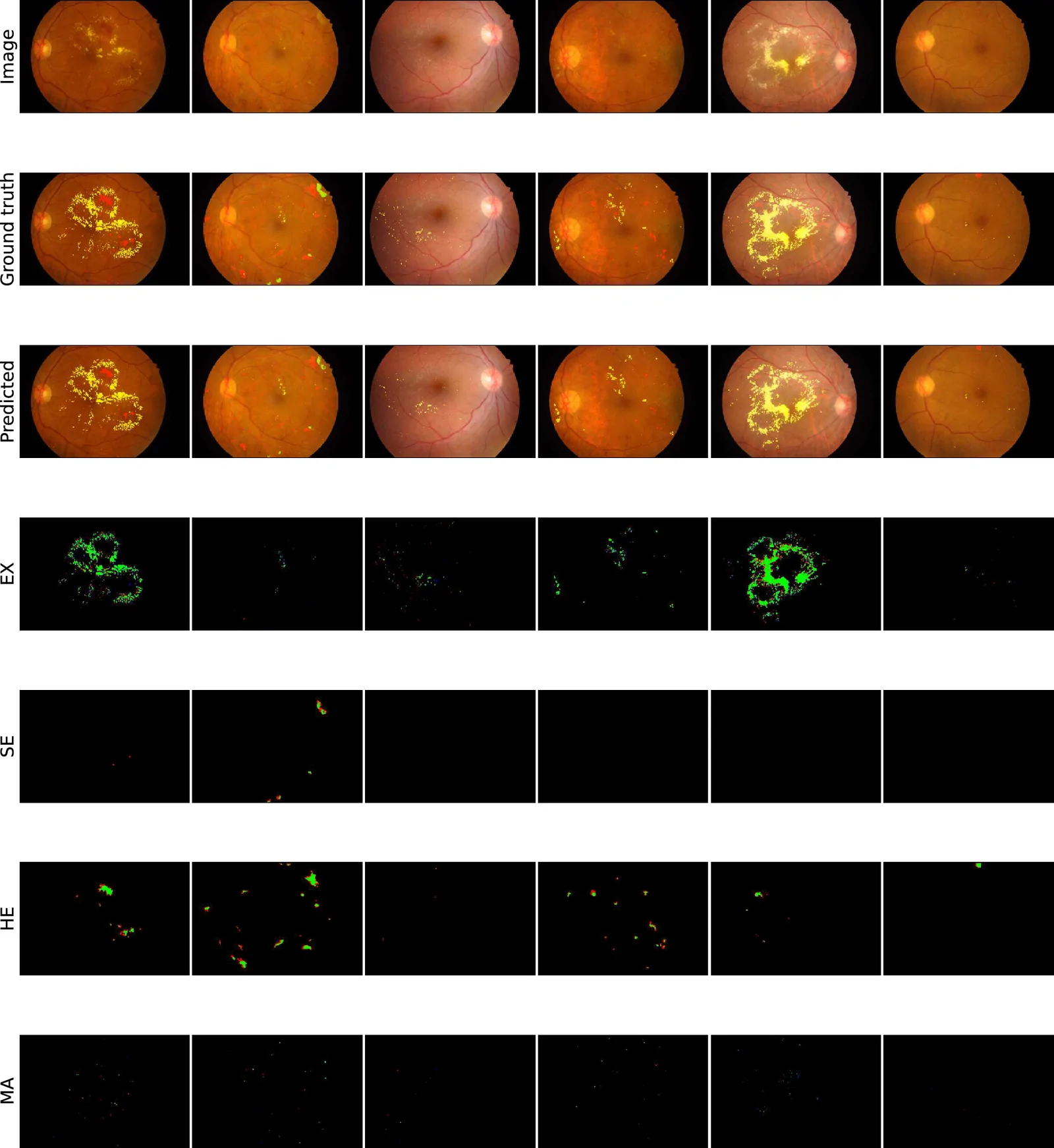
Segmentation results on DDR test set. The first row shows the original images. The second row shows the corresponding ground truths. The third row shows the segmentation results. The yellow, green, red, and orange pixels denote the EX, SE, HE, and MA lesions, respectively. Rows four to seven show the segmentation results for each lesion type individually. In these images, green pixels represent true positives, red pixels indicate false negatives, and blue pixels denote false positives.

Some samples of segmentation results from DDR and IDRiD test sets using the optimal configuration, referred to as DDFU-Net, are shown in Figs. 3 and 4, where the original RGB images, corresponding ground truths, and our results are listed on the first, second, and third rows, respectively. For a better visualization of fine details, zoomed-in comparisons of relevant lesions can be found in the Supplementary Material Figs. S1 and S2.

## Discussion

### Overall Segmentation Performance

This work presents a novel deep-learning framework for retinal lesion segmentation, introducing an asymmetric dense U-Net architecture. Our results establish a performance hierarchy among the structural variants, demonstrating the superiority of decoder-heavy models over symmetric structures, while encoder-heavy designs proved to be the least effective. Through extensive experimentation, we have identified the optimal configuration, DDFU-Net, comprising five dense blocks in the encoder and seven dense blocks in the decoder within a multi-task learning framework.

Traditional symmetric U-Net structures often suffer from over-compression [18], leading to the loss of fine-grained features essential for segmenting small lesions. Regarding symmetric architectures ($N_e = N_d$), Tables 2 and 3 indicate that performance plateaus at five blocks, after which deeper networks suffer from degradation. Our experiments show that exceeding five blocks with an input resolution of 512×512 pixels results in inferior performance. This suggests that a symmetrical up-sampling path is insufficient to fully recover the high-frequency spatial details of micro-lesions lost during down-sampling.

Furthermore, encoder-heavy configurations ($N_e> N_d$) yielded the lowest IoU and Dice scores (Tables 4 and 5). This supports our theoretical analysis regarding the truncated inverse operator (Eq. 4), where excessive encoder depth creates an information bottleneck, while a shallow decoder lacks the structural capacity to invert. Additionally, these configurations result in excessive model complexity. For instance, the 7-5 configuration requires over 1.2 billion parameters, whereas the optimal 5-7 configuration utilizes only 77 million due to the geometric expansion of filters in the encoder. Moreover, the inferior performance of encoder-heavy models suggests that the excessive number of parameters to optimize leads to poorer outcomes.

Conversely, the decoder-heavy configuration (specifically DDFU-Net with $N_e=5, N_d=7$) achieved the global optimum across both datasets while maintaining high parameter efficiency. By limiting encoder depth while expanding the decoder, this design mitigates over-compression and enables a smoother, progressive restoration of high-resolution maps. Crucially, these results confirm that the decisive factor is allocating computational resources to the non-linear refinement operator $\Phi _{\text {Refine}}$ (Eq. 6). This extended decoding phase maximizes the capacity of the network to approximate the inverse mapping $\mathcal {P}^{-1}$ (Eq. 9), ensuring that detailed structures like microaneurysms and hemorrhages are accurately reconstructed. Consequently, this design is particularly beneficial for retinal lesion segmentation, where small lesions and high inter-class similarities pose significant challenges.

Finally, the multi-task learning setup offers significant advantages in both accuracy and computational efficiency. Multi-task training demonstrates superior performance compared to single-task training, likely because the strategy leverages shared lesion features to enhance the performance of each sub-task [39]. Regarding efficiency, inference using the proposed DDFU-Net takes approximately 0.71 s per image on our hardware. In contrast, a single-task approach requires evaluating four separate models, effectively quadrupling the inference time and resource consumption.

Tables 6 and 7 show that, overall, our model achieves the highest performance compared to state-of-the-art methods on both datasets. To complement the quantitative analysis of the performance, Figs. 3 and 4 illustrate the segmentation results on the DDR and IDRiD test sets. The first row presents the original images, and the second row shows the corresponding ground truths. The third row contains the segmentation results produced by DDFU-Net, displaying the segmentation outcomes for each lesion type: EX (yellow), SE (green), HE (red), and MA (orange). Last rows show the segmentation for each lesion type individually differentiating between true positives (green pixels), false negatives (red pixels), and false positives (blue pixels). Furthermore, to enable a more detailed inspection of small lesions and boundary definitions, Supplementary Figs. S1 and S2 provide paired visualizations containing the full fundus image alongside a zoomed-in view of the specific regions of interest.

The segmentation of EX reveals a generally high concordance with the ground truth. On both datasets, it maintains accuracy comparable to existing methods, demonstrating its ability to detect and delineate these lesions accurately. Notably, on the DDR dataset, DDFU-Net achieves the best performance compared to state-of-the-art methods, with an AUPR of 64.71%, an IoU of 45.15%, and a Dice score of 60.42%. On IDRiD, it attains an AUPR of 66.13%, an IoU of 58.21%, and a Dice score of 73.58%. While the quantitative results for EX do not surpass all state-of-the-art methods on IDRiD, the qualitative analysis in Fig. 3, and the zoomed details in Supplementary Fig. S1, reveals that true-positive regions (green pixels) effectively cover most of the areas identified in the ground truth. The segmentation results suggest that DDFU-Net is capable of capturing fine details, particularly at lesion edges.

DDFU-Net shows outstanding performance in segmenting SE, achieving the highest IoU (46.73%) and Dice (63.69%) on the DDR dataset, as well as remarkable results on the IDRiD dataset with IoU (79.63%) and Dice (88.66%). The green pixels denote accurate detection but are less prevalent than in the EX segmentation. There is an increase in red pixels (false negatives), which points to challenges in precisely identifying SE. This could be attributed to the subtle and diffuse nature of SE, making them harder to segment accurately.

The segmentation of HE and MA poses notable challenges due to their small size and visual similarity. Despite this, DDFU-Net achieves the highest Dice score for HE on both datasets (50.66% on DDR and 79.87% on IDRiD), indicating a strong capacity for accurate segmentation. True positives (green) are present but often interspersed with red (false negatives) and blue pixels (false positives). The increased presence of false negatives suggests that the model occasionally misses HE, which could be due to their varying appearance and size, making them less distinguishable from the background and MAs. On the DDR dataset, DDFU-Net achieves the best AUPR (42.49%) for MA. However, Figs. 3 and 4 reveal that MA segmentations often correspond to HE masks in the ground truth, indicating a high rate of misclassification. This misclassification results in false positives (blue) in the MA segmentation and false negatives (red) in the HE segmentation, as shown in the last two rows of Supplementary Figs. S1 and S2. The similar visual characteristics of small HEs and MAs, such as their small size and round shape, contribute to this confusion, making it difficult for the model to accurately differentiate between them.

Overall, the model demonstrates varying levels of accuracy across different lesion types in the DDR and IDRiD datasets. The segmentation of EX and SE shows high accuracy, whereas the detection of HE and MA poses more challenges due to their similarity.

**Table 6:** Comparison results on DDR test set

Methods	AUPR					IoU					Dice				
	mAUPR	EX	HE	SE	MA	mIoU	EX	HE	SE	MA	mDice	EX	HE	SE	MA
L-seg [7]	32.08	55.46	35.86	26.48	10.52	–	–	–	–	–	–	–	–	–	–
Dual-PSPNet [40]	–	54.24	–	–	–	–	39.85	–	–	–	–	56.99	–	–	–
RTNet [3]	33.62	56.71	36.56	29.43	11.76	–	–	–	–	–	–	–	–	–	–
HED [41]	42.97	61.40	43.19	46.68	20.61	27.17	39.50	27.09	29.46	12.63	41.79	56.63	42.61	45.50	22.43
DNL [42]	40.14	56.05	47.81	42.01	14.71	24.33	36.39	27.15	25.33	8.46	38.02	53.36	42.71	40.40	15.60
Deeplabv3+ [16]	42.34	62.32	40.79	41.83	24.39	26.47	41.44	23.44	26.46	14.55	40.95	58.59	37.97	41.83	25.40
PSPNet [43]	39.23	57.04	42.71	42.32	14.85	24.31	37.31	24.51	26.64	8.75	37.97	54.35	39.37	42.08	16.09
SPNet [44]	31.91	44.10	38.22	32.93	12.37	16.47	24.19	13.76	20.55	7.38	27.66	38.78	24.13	34.00	13.74
HRNetV2 [45]	45.21	61.55	45.68	46.91	26.70	28.84	41.82	29.01	28.94	15.60	43.95	58.98	44.96	44.86	26.99
Swin-base [17]	46.72	62.71	54.39	46.12	23.67	30.07	42.64	*33.82*	30.62	13.19	45.10	59.79	*50.53*	46.77	23.31
Twins-SVT-B [46]	46.11	59.71	49.96	52.72	22.03	29.28	39.70	29.08	*36.24*	12.07	44.15	56.83	45.04	*53.19*	21.54
M2MRF-A [12]	*49.94*	*64.17*	54.20	*53.19*	28.21	*31.16*	43.35	30.03	35.22	16.06	*46.60*	60.47	46.18	52.10	27.67
M2MRF-B [12]	49.42	63.88	*55.47*	50.01	28.33	30.41	43.06	30.56	32.08	15.95	45.77	60.20	46.81	48.58	27.51
M2MRF-C [12]	48.94	63.59	54.43	49.35	28.38	30.09	43.49	29.17	31.39	*16.31*	45.40	*60.62*	45.16	47.78	*28.04*
M2MRF-D [12]	49.25	*64.17*	54.72	49.64	*28.46*	30.27	*44.04*	29.28	31.60	16.15	45.57	**61**.**15**	45.29	48.02	27.81
Proposed method (DDFU-Net)	**54**.**86**	**64**.**71**	**56**.**56**	**55**.**67**	**42**.**49**	**36**.**96**	**45**.**15**	**33**.**93**	**46**.**73**	**22**.**01**	**52**.**24**	60.42	**50**.**66**	**63**.**69**	**34**.**20**

Results marked in bold are the highest ones while those in italic are the second highest ones

### Comparison to State-of-the-Art Segmentation Methods on DDR Dataset

We evaluated DDFU-Net against 12 state-of-the-art segmentation methods: L-seg [7], Dual-PSPNet [40], RTNet [3], HED [41], DNL [42], Deeplabv3+ [16], PSPNet [43], SPNet [44], HRNetV2 [45], Swin-base [17], Twins-SVT-B [46], and M2MRF [12]. Comparative results are listed in Table 6.

Our model shows improvements in both mean and category-specific metrics. Notably, for all types of lesions, high values of AUPR and IoU were observed. When compared to the top-performing state-of-the-art framework M2MRF [12], our proposed architecture demonstrates consistent superiority. For MAs, which are particularly small and difficult to detect, DDFU-Net achieved an absolute improvement of 14.03% in AUPR compared to M2MRF-D [12]. This improvement may be due to our architecture avoiding uniform subsampling, thus preserving more informative activations for tiny lesions. In the case of SEs, which appear similarly to EXs, our model achieved an absolute increase of 0.54% in AUPR compared to the best previous architecture M2MRF-B. DDFU-Net is able to reassemble features within large regions, thereby exploiting long-term dependencies that enhance feature representation and facilitate classification. Additionally, DDFU-Net outperforms the best M2MRF-A variant by absolute margins of 4.92%, 5.80%, and 5.64% in mAUPR, mIoU, and mDice, respectively.

Compared to the most recent transformer-based methods, Swin-base [17] and Twins-SVT-B [46], DDFU-Net also shows superior performance. Specifically, DDFU-Net surpasses Swin-base [17] by absolute margins of 8.14% in mAUPR, 6.89% in mIoU, and 7.14% in mDice. Likewise, against Twins-SVT-B [46], our model achieves absolute gains of 8.75% in mAUPR, 7.68% in mIoU, and 8.09% in mDice.

Segmentation results from DDFU-Net in some images from the DDR test set are illustrated in Figure 3. The first row shows the original RGB images, the second row presents the corresponding ground truths, the third row displays our results and the last rows show the segmentation for each lesion type individually differentiating between true positives, false negatives, and false positives. In the first column, we observe that the predicted segmentations for EXs appear to adapt more accurately to the lesion boundaries compared to the original segmentations, aligning more closely with the ground truth. The segmentations provided in the dataset appear to be coarsely annotated, while our results more accurately reflect pigmentation changes caused by the lesions. This highlights the challenges inherent in pixel-level annotation, where even expert annotations may lack precision due to the complexity of delineating lesion boundaries. These inherent limitations in the annotation process may be causing such discrepancies with the ground truth. The correct segmentation of SE can be seen in columns 3 and 6, though it appears visually similar to EX. The segmentation of HEs and MAs shows that red pixels (false negatives) in HEs often correspond to blue pixels (false positives) in MAs, indicating that the model occasionally misclassifies small HEs as MAs.

**Table 7:** Comparison results on IDRiD test set

Methods	AUPR					IoU					Dice				
	mAUPR	EX	HE	SE	MA	mIoU	EX	HE	SE	MA	mDice	EX	HE	SE	MA
VRT [10]	64.69	71.27	68.04	*69.95*	*49.51*	–	–	–	–	–	–	–	–	–	–
PATech [10]	–	*88.50*	64.90	–	47.70	–	–	–	–	–	–	–	–	–	–
iFLYTEK [10]	64.84	87.41	55.88	65.88	**50**.**17**	–	–	–	–	–	–	–	–	–	–
L-seg [7]	65.15	79.45	63.74	71.13	46.27	–	–	–	–	–	–	–	–	–	–
Dual-PSPNet [40]	–	77.48	–	–	–	–	60.95	–	–	–	–	75.74	–	–	–
RTNet [3]	**70**.**76**	**90**.**24**	*68.80*	*75.02*	*48.97*	–	–	–	–	–	–	–	–	–	–
HED [41]	63.94	80.81	66.41	68.09	40.45	46.66	64.74	47.43	50.38	24.07	62.18	78.60	64.33	67.00	38.81
DNL [42]	59.09	75.12	64.04	64.73	32.48	42.28	57.67	44.80	47.03	19.61	57.94	73.15	61.87	63.96	32.78
Deeplabv3+ [16]	63.19	81.93	64.66	63.04	43.14	45.21	66.10	44.90	44.39	25.45	60.90	79.60	61.96	61.48	40.57
PSPNet [43]	58.73	75.21	63.36	63.65	32.71	41.70	57.78	43.71	45.81	19.50	57.38	73.24	60.81	62.83	32.63
SPNet [44]	50.25	64.61	54.11	52.14	30.14	30.30	44.40	28.50	33.58	14.72	45.26	61.45	44.33	49.59	25.66
HRNetV2 [45]	65.01	82.09	65.50	68.68	43.76	47.52	*66.57*	45.56	50.99	26.98	63.14	*79.93*	62.58	67.53	42.49
Swin-base [17]	64.48	81.34	66.57	64.91	45.10	47.76	66.26	48.36	47.54	28.86	63.53	79.71	65.19	64.43	44.79
Twins-SVT-B [46]	63.84	80.09	63.12	68.86	43.27	47.07	64.68	44.91	*51.76*	26.92	62.79	78.56	61.98	*68.19*	42.42
M2MRF-A [12]	66.48	82.04	67.80	68.23	47.86	49.07	66.16	48.87	50.03	31.23	64.89	79.64	65.66	66.68	47.59
M2MRF-B [12]	66.00	81.98	67.41	66.68	47.91	48.56	66.07	48.58	48.16	31.42	64.45	79.57	65.39	65.01	47.81
M2MRF-C [12]	*67.24*	82.16	68.69	69.32	48.80	*49.94*	66.46	*49.72*	51.43	**32**.**13**	*65.71*	79.85	*66.42*	67.92	**48**.**63**
M2MRF-D [12]	66.66	82.29	66.94	69.00	48.43	49.36	**66**.**62**	48.04	50.98	*31.81*	65.15	**79**.**97**	64.88	67.50	*48.26*
Proposed method (DDFU-Net)	66.69	66.13	**75**.**09**	**86**.**34**	39.20	**57**.**31**	58.21	**66**.**49**	**79**.**63**	24.91	**69**.**93**	73.58	**79**.**87**	**88**.**66**	39.60

Results marked in bold are the highest ones while those in italic are the second highest ones

### Comparison to State-of-the-Art Segmentation Methods on IDRiD Dataset

We evaluated our architecture, DDFU-Net, against 15 state-of-the-art approaches, including VRT, PATech, iFLYTEK, L-seg [7], Dual-PSP [40], RTNet [3], HED [41], DNL [42], Deeplabv3+ [16], PSPNet [43], SPNet [44], HRNetV2 [45], M2MRF [12], Swin-base [17], and Twins-SVT-B [46]. Comparative results are shown in Table 7.

Our model shows improvements in both mean and category-specific metrics. For instance, we surpassed VRT and iFLYTEK, the top two methods in the 2018 ISBI grand challenge ‘Diabetic Retinopathy-Segmentation and Grading’ [10], by absolute margins of 2.00% and 1.85% in mAUPR, respectively. These methods used a separate CNN for segmenting each lesion type. However, RTNet [3] significantly outperforms our model in terms of mAUPR. This is likely because RTNet leverages two additional datasets, DRIVE [47] and STARE [48], which include pixel-level vessel annotations, enhancing its performance on lesion segmentation. In contrast, our model only uses the training set of IDRiD [20]. Despite this, DDFU-Net achieves remarkably higher precision in detecting specific lesions compared to multi-stage frameworks. For SEs, our model outperforms the best M2MRF variant (M2MRF-C) with an absolute AUPR improvement of 17.02%. Furthermore, in terms of segmentation quality metrics, DDFU-Net surpasses M2MRF-C [12] by absolute margins of 7.37% and 4.22% in mIoU and mDice, respectively. For transformer-based methods, DDFU-Net surpasses Swin-base [17] by absolute differences of 2.21%, 9.55% and 6.4% in mAUPR, mIoU, and mDice, respectively. Similarly, against Twins-SVT-B [46], DDFU-Net achieves absolute improvements of 2.85%, 10.24%, and 7.14% in mAUPR, mIoU, and mDice, respectively.

Some segmentation results from DDFU-Net are illustrated in Fig. 4, where the original RGB images, corresponding ground truths, and our results are displayed in the first, second, and third rows, respectively. The model correctly identifies SEs marked by green pixels in the second and sixth columns, despite the similar appearances of SEs and EXs, which often lead to confusion. Regarding HEs and MAs, there is an increased presence of red pixels (false negatives). This could be due to the inherent difficulty in distinguishing HEs and MAs, both of which are dark red lesions with similar appearances. Overall, the segmentation results closely match the ground truth, and in most cases, the DDFU-Net segmentation is contained within the annotated regions of the dataset. This suggests that the model may demonstrate higher precision in segmentation, detecting subtle changes in the image.

### Limitations and Future Work

This study presents some limitations that should be pointed out. First, DDR and IDRiD publicly available datasets contain a limited number of annotated images, which may be enough for traditional methods but not enough for deep neural networks. Following this direction, our future work includes designing a data augmentation deep-learning model to generate synthetic images based on lesion masks. This model will enable the creation of a synthetic fundus image dataset containing thousands of annotated images. Additionally, future work will focus on exploring the mutual dependencies between lesion segmentation and eye disease grading tasks. We aim to develop a deep multi-task learning framework for joint lesion segmentation and eye disease classification, enhancing the performance and utility of our proposed model. Finally, it is important to acknowledge that the current evaluation is limited to datasets specifically annotated for diabetic retinopathy. In clinical practice, patients may present with other retinal pathologies that manifest lesions visually similar to those found in diabetic retinopathy. A primary example is drusen, a hallmark of age-related macular degeneration, which shares significant characteristics with hard exudates. Distinguishing between these confounding lesions is essential to avoid misdiagnosis and ensure accurate disease grading. Therefore, future work will focus on validating DDFU-Net against broader datasets containing diverse retinal diseases, specifically testing its ability to discriminate between hard exudates and drusen.

## Conclusions

Our study introduces a novel network, DDFU-Net, an asymmetric dense U-Net designed for multi-task lesion segmentation. This network effectively addresses the challenge of segmenting different lesions of varying shapes and sizes. Comprehensive evaluations demonstrate that DDFU-Net significantly enhances performance over existing state-of-the-art CNN-based methods [16, 41–45] and leading transformer-based segmentation methods [17, 46]. These results suggest that the proposed method can be used to segment common lesions associated with multiple retinal disorders, including EX, SE, HE, and MA, contributing to more accurate diagnostic processes.

## Supplementary Information

Below is the link to the electronic supplementary material.Supplementary file 1 (pdf 657 KB)
