# Supplementary material for: DDFU-Net: A Deep Decoder-Focused U-Net Model for Retinal Lesion Segmentation
Source: Ann Biomed Eng. 2026 Mar 12;54(9):3231–51. doi: 10.1007/s10439-026-04032-w (PMC13522056; doi:10.1007/s10439-026-04032-w)
Supplement: Supplementary file 1 [file 10439_2026_4032_MOESM1_ESM.pdf]

Supplementary Materials

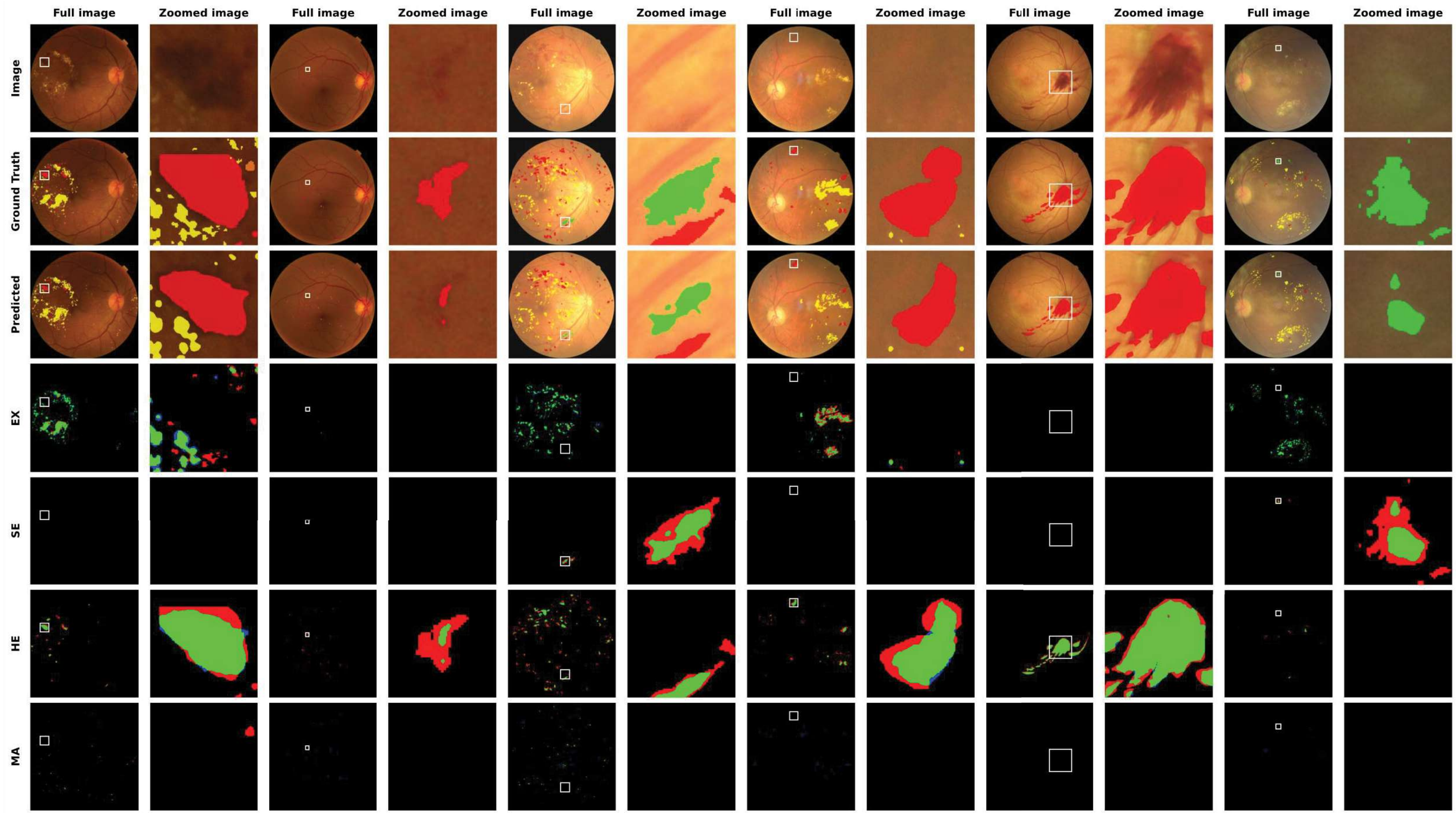

Fig S1: Segmentation results on the DDR test set. The first row shows the original images. The second and third rows present the ground truth and model predictions, respectively, using a lesion-specific color map: yellow (EX), green (SE), red (HE), and orange (MA). Rows 4 through 7 display the results for each lesion type individually. In these rows, the color coding reflects performance: green pixels represent true positives, red pixels indicate false negatives, and blue pixels denote false positives. Each example is presented in paired columns: the full fundus image on the left and a zoomed view on the right, corresponding to the region outlined by the white box.

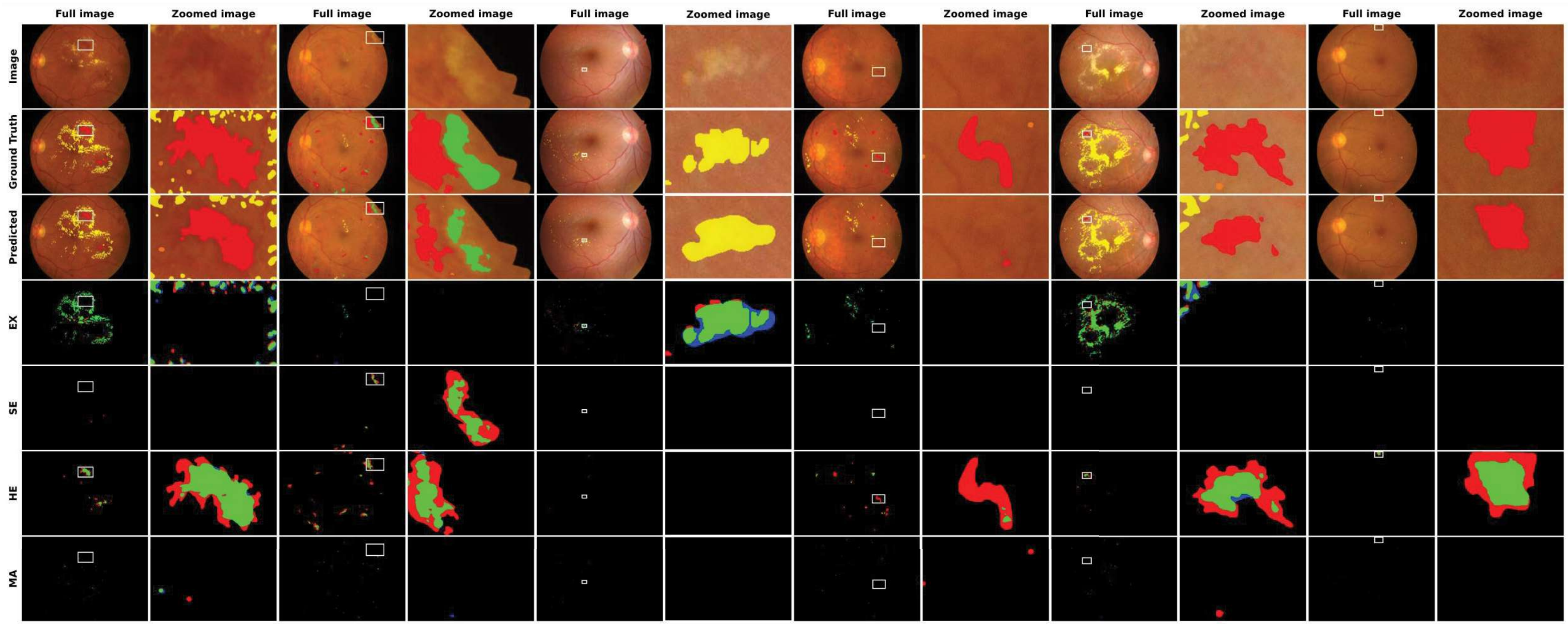

Fig S2: Segmentation results on the IDRiD test set. The first row shows the original images. The second and third rows present the ground truth and model predictions, respectively, using a lesion-specific color map: yellow (EX), green (SE), red (HE), and orange (MA). Rows 4 through 7 display the results for each lesion type individually. In these rows, the color coding reflects performance: green pixels represent true positives, red pixels indicate false negatives, and blue pixels denote false positives. Each example is presented in paired columns: the full fundus image on the left and a zoomed view on the right, corresponding to the region outlined by the white box.
